# Eculizumab therapy on a patient with co‐existent lupus nephritis and C3 mutation‐related atypical haemolytic uremic syndrome: a case report

**DOI:** 10.1186/s12882-021-02293-2

**Published:** 2021-03-10

**Authors:** Mi Jung Kim, Haekyung Lee, Yon Hee Kim, So Young Jin, Hee-Jin Kim, Doyeun Oh, Jin Seok Jeon

**Affiliations:** 1 Asan Yuri Hospital, 179 Dogomyeon-ro, Dogo-myeon, 31551 Asan-si, Chungcheongnam-do Republic of Korea; 2 Division of Nephrology, Department of Internal Medicine, Soonchunhyang University Seoul Hospital, 59 Daesagwan-ro, Yongsan-gu, 04401 Seoul, Republic of Korea; 3 Department of Pathology, Soonchunhyang University Seoul Hospital, 59 Daesagwan-ro, Yongsan-gu, 04401 Seoul, Republic of Korea; 4 Department of Laboratory Medicine & Genetics, Samsung Medical Center, Sungkyunkwan University School of Medicine, 81 Irwon-ro, Gangnam-gu, 06531 Seoul, Republic of Korea; 5 Department of Internal Medicine, CHA Bundang Medical Center, CHA University, 59 Yatapro, Bundang- gu, 13496 Seonganm-si, Gyeonggi Korea

**Keywords:** Atypical haemolytic uremic syndrome, Case report, Eculizumab, Lupus nephritis, Thrombotic microangiopathy

## Abstract

**Background:**

Thrombotic microangiopathy (TMA), a rare but serious complication of systemic lupus erythematosus (SLE), is associated with poor outcomes to conventional immunosuppressive therapy. Recently, eculizumab, a humanised monoclonal antibody that blocks the complement factor 5, has been known to effectively treat atypical haemolytic uremic syndrome (aHUS). Here, we report a case of aHUS co-existing with lupus nephritis that was successfully treated with eculizumab.

**Case presentation:**

A 23-year-old man presented with abdominal pain and diarrhoea. Initial laboratory tests have shown thrombocytopaenia, microangiopathic haemolytic anaemia, and acute kidney injury. Immunologic tests were consistent with SLE. Kidney biopsy have revealed lupus nephritis class IV-G with TMA. Genetic analysis have shown complement C3 gene mutations, which hints the co-existence of lupus nephritis with aHUS, a form of complement-mediated TMA. Although initial treatment with haemodialysis, plasma exchange, and conventional immunosuppressive therapy (steroid and cyclophosphamide) did not appreciably improve kidney function and thrombocytopaenia, the patient was able to respond to eculizumab therapy.

**Conclusions:**

Due to the similar features of TMA and SLE, clinical suspicion of aHUS in patients with lupus nephritis is important for early diagnosis and prompt management. Timely administration of eculizumab should be considered as a treatment option for aHUS in lupus nephritis patients to yield optimal therapeutic outcomes.

## Background

Atypical haemolytic uremic syndrome (aHUS), also known as complement-mediated thrombotic microangiopathy (TMA), is characterised by microangiopathic haemolytic anaemia, thrombocytopaenia, and acute kidney injury [1]. It is known that primary aHUS is associated with genetic mutations in the alternative complement pathway, while secondary HUS is caused by various aetiologies, including drugs, infection, cancer, and autoimmune diseases [2].

Systemic lupus erythematosus (SLE) is an autoimmune disease that involves multiple systems. Furthermore, SLE is an independent risk factor for the development of TMA and the prognosis of lupus nephritis worsen if there is a co-existent TMA despite conventional immunosuppressive agents [3]. Eculizumab, a terminal complement inhibitor, has been reported to show favourable therapeutic response for primary aHUS [4]. However, the use of eculizumab for treatment of secondary HUS remains controversial [5, 6]. In this report, we describe a patient diagnosed with concurrent C3 mutation-related aHUS and lupus nephritis who was successfully treated with eculizumab. This complement inhibitor could be a potential treatment for patients with co-existent SLE and complement-mediated TMA that are refractory to conventional immunosuppressive therapies.

## Case presentation

A 23-year-old man was transferred from a local hospital due to diarrhoea, thrombocytopaenia, and azotaemia after transfusion with red blood cells to correct anaemia. Three days prior to admission, he developed general malaise, abdominal pain, and diarrhoea. He denied any cutaneous and musculoskeletal symptoms, and his past medical history and family history were unremarkable. On the day of admission, he presented with hypertension (170/110 mmHg). Physical examination has shown pitting oedema in the lower extremities and direct tenderness in the right upper quadrant of the abdomen. Table 1 summarises the initial laboratory findings. The patient presented with leukopenia (white blood cell count 3,900/µL), haemolytic anaemia (haemoglobin 9.7 g/dL, haematocrit 29.2 %, and haptoglobin < 10 mg/dL), thrombocytopaenia (platelet 41,000/µL), and azotaemia (blood urea nitrogen 52.9 mg/dL and serum creatinine 2.8 mg/dL). The patient had decreased total protein (4.9 g/dL) and albumin (2.1 g/dL). He showed elevated levels of total cholesterol (202 mg/dL) and triglyceride (236 mg/dL). In addition, the lactate dehydrogenase level was elevated (457 U/L), whereas aspartate/alanine transaminase levels (14/8 U/L) were within normal values. In terms of serum electrolytes, the uric acid level (9.4 mg/dL) was elevated while the sodium (142 mmol/L) and potassium (5.2 mmol/L) levels were normal.

**Table 1:** Clinical course of the patient

Variables	Normal values	On admission	Follow-up (after 32 cycles)
**Haematological parameters**			
Haemoglobin (g/dL)	13–17	9.7	13.7
Schistocytes	Not found	Found	Not found
Platelets (/uL)	130,000–450,000	41,000	149,000
Leukocytes (/uL)	4000–10,000	3900	4200
LDH (U/L)	140–280	457	154
Haptoglobin (mg/dL)	50–220	< 10	119
**Renal parameters**			
BUN (mg/dL)	6–20	52.9	13.0
Creatinine [mg/dL] (eGFR [ml/min])	0.5–1.2	2.80 (30.42)	1.10 (93.46)
Protein (g/dL)	6.4–8.3	4.9	6.4
Albumin (g/dL)	3.5–5.2	2.1	4.3
Haematuria (/HPF)	0–2	Many	10–19
UACR (mg/g)	0–30	3607.7	206.4
**Immunological parameters**			
Antinuclear Ab	Neg	Speckled (1:5120), Cytoplasmic (1:2560)	Speckled (1:1280)
Anti-ds DNA Ab (IU/mL)	0–7	1228.3	11.6
Anti-nucleosome Ab	Neg	1+	Neg
C3 (mg/dL)	90–180	29	89
C4 (mg/dL)	10–40	3	18
Lupus anticoagulant	Neg	Neg	
Anti-cardiolipin IgM/IgG	Neg/Neg	Neg/Neg	
Anti-β2 glycoprotein 1 IgM/IgG	Neg/Neg	Neg/Pos	

*BUN *Blood urea nitrogen, *eGFR *Estimated glomerular filtration rate, *HPF *High-power field, *LDH *Lactate dehydrogenase, *Neg *Negative, *Pos *Positive, *UACR *Urine albumin-to-creatinine ratio

A peripheral blood smear has revealed the presence of schistocytes. Urinalysis has shown proteinuria (4+) with a urine albumin-to-creatinine ratio of 3607.7mg/g and haematuria (many red blood cells/high power fields). Tests for hepatitis B surface antigen and antibody to hepatitis C virus were all negative. Below the normal limits, the serum complement C3 level and C4 level were 29 mg/dL and 3 mg/dL, respectively. Antinuclear antibodies and anti-double-stranded DNA were positive. Negative results were obtained for lupus anticoagulant, anti-cardiolipin IgM/IgG, and anti-β2 glycoprotein 1 IgM, whereas a positive result was obtained for anti-β2 glycoprotein I IgG. ADAMTS13 (a disintegrin and metalloproteinase with thrombospondin type 1 motif, member 13) activity was 90 %. Stool examination did not show any Shiga toxin-producing *Escherichia coli* O157:H7.

**Fig. 1:**
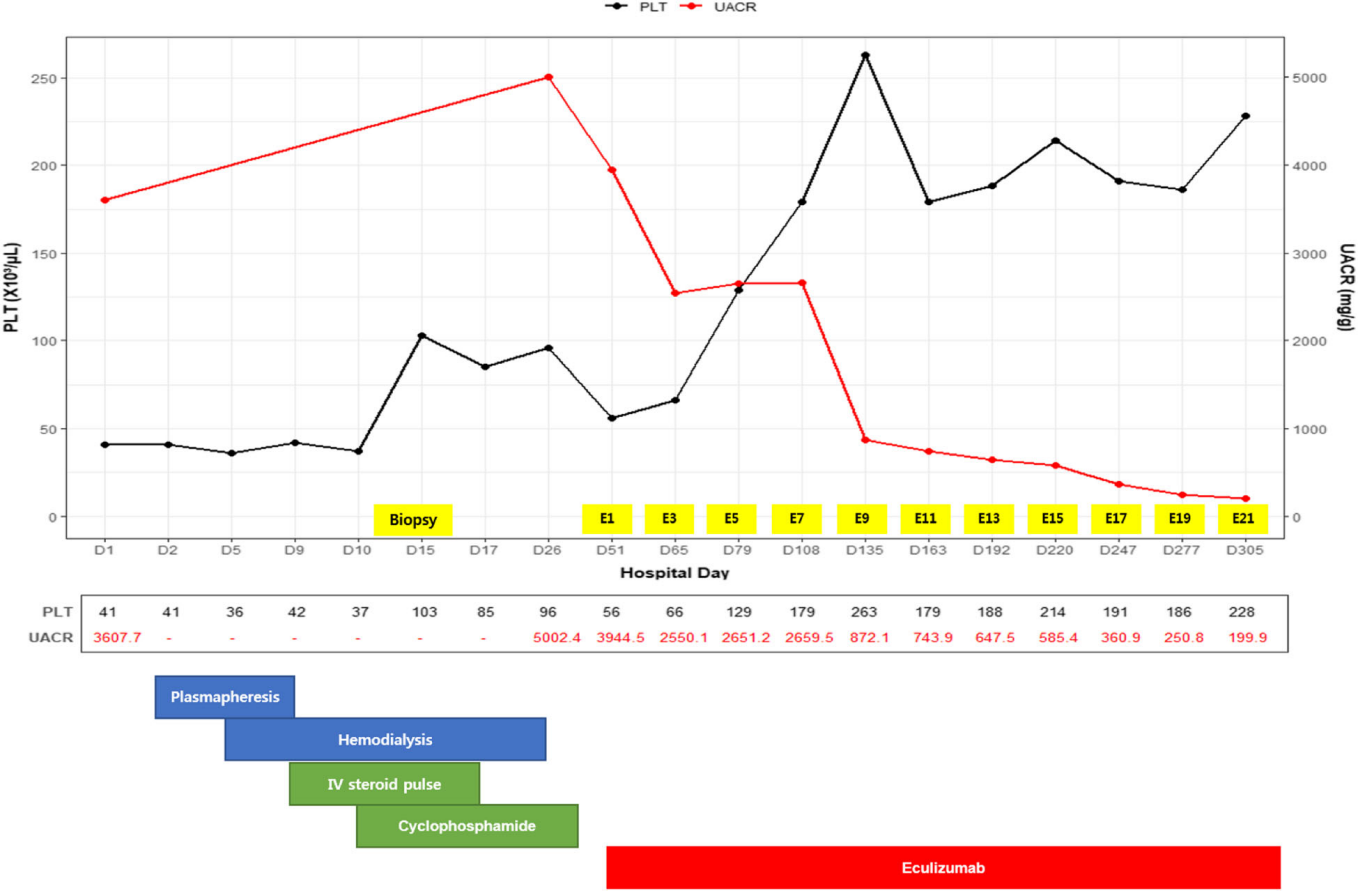
Clinical course and key events. Abbreviations: E, eculizumab; IV, intravenous; PLT, platelet; UACR, urine albumin-to-creatinine ratio

Due to the clinical manifestations and laboratory findings, diagnoses of TMA and SLE were made. The time sequence of key events and the clinical course are presented in Fig. 1. Plasmapheresis was started to treat SLE and TMA. Haemodialysis was initiated due to oliguria and volume overload. The patients received pulse IV methylprednisolone and 500 mg cyclophosphamide every 2 weeks. Although thrombocytopaenia improved, the platelet count (103,000/µL) and kidney function parameters (blood urea nitrogen 59.7 mg/dL, serum creatinine 4.95 mg/dL) were not within normal limits.

**Fig. 2:**
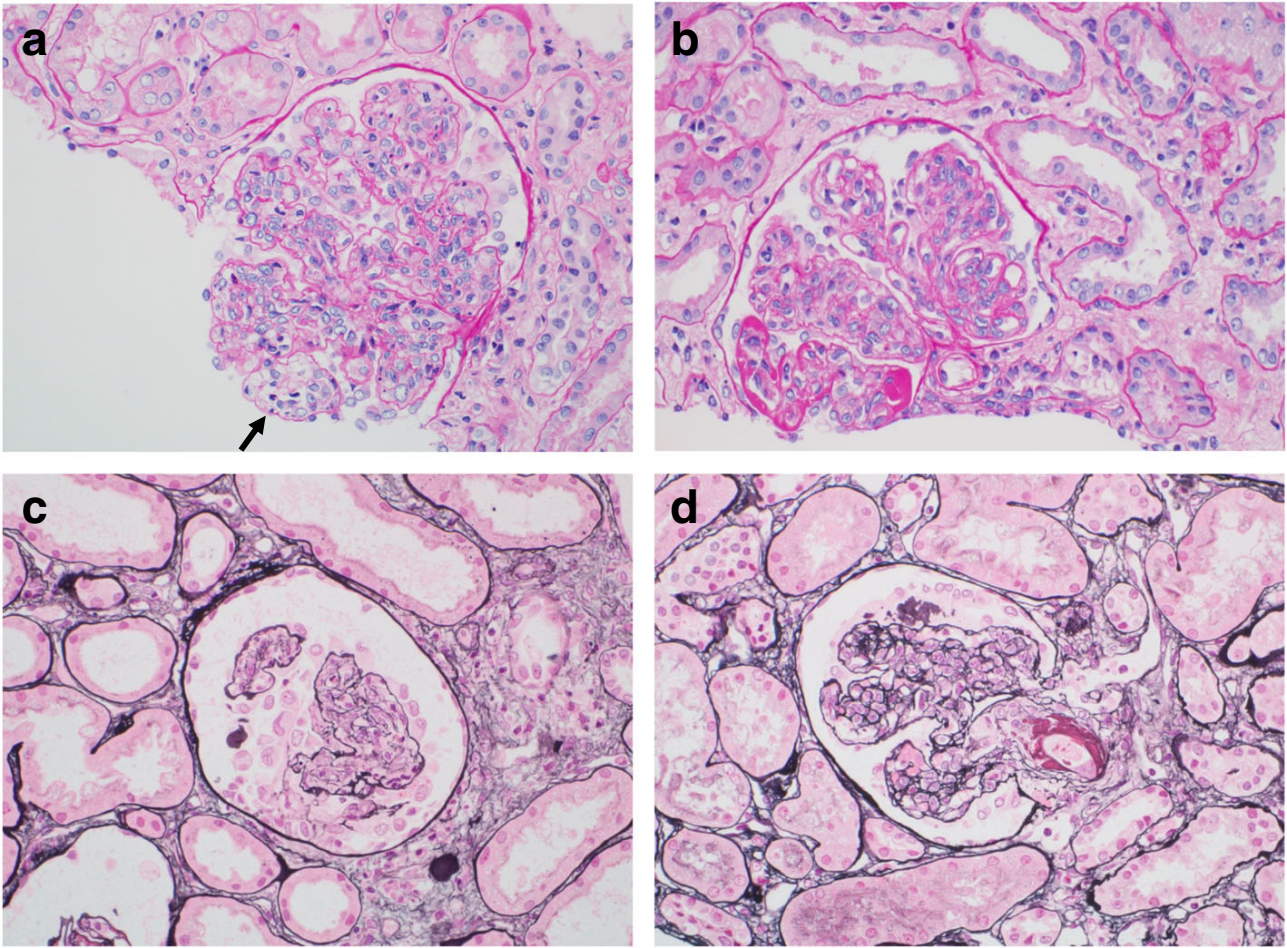
Pathological findings of kidney biopsy. **a** Light microscopy, Haematoxylin and eosin staining (H&E). Lobular accentuation with panhypercellularity and multiple layers of capillary wall with focal segmental mesangiolysis (arrow). **b** Light microscopy, H&E. Glomerulus showing marked thickened capillary loop (wire loop lesion). **c** Light microscopy, silver staining. Cellular crescent in glomerulus. **d** Light microscopy, silver staining. Afferent arterioles affected by fibrinoid necrosis

On the 15th hospital day, ultrasound-guided percutaneous kidney biopsy was performed. Light microscopy of renal biopsy specimens has shown diffuse proliferative lupus nephritis, class IV-G (active) with mesangiolysis and arteriolar fibrin thrombi indicative of TMA (Fig. 2). Immunofluorescence staining has shown a “full-house” pattern (positive for IgG, IgA, IgM, C3, and C1q) in the mesangium and peripheral capillary wall. Based on these renal biopsy findings, a diagnosis of lupus nephritis with TMA was confirmed.

Next-generation sequencing and whole exon sequencing have revealed heterozygous mutation c.1685 C > T (p.ser562Leu) of the C3 gene. The presence of C3 mutation and incomplete response to conventional immunosuppression (Fig. 1) are consistent with diagnosis of co-existent lupus nephritis and aHUS (complement-mediated TMA). Haemodialysis and cyclophosphamide therapy was discontinued after 10 sessions and 3 administrations, respectively. On the 51st hospital day, treatment was shifted to intravenous eculizumab at an initial dose of 900 mg for four times a week escalated to 1,200 mg every two weeks thereafter. After 32 cycles, there was complete resolution of haemolytic anaemia (haemoglobin 13.7 g/dL, haematocrit 41.8 %, and haptoglobin 119 mg/dL), thrombocytopaenia (platelet count 149,000/µL), and azotaemia (blood urea nitrogen 13.0 mg/dL, serum creatinine 1.10 mg/dL). The lactate dehydrogenase level normalized (154 U/L) and the Urine albumin-to-creatinine ratio (206.4 mg/g) was greatly improved (Table 1). At the time of writing this report, the patient was taking a 10-mg dose of a corticosteroid daily together with eculizumab therapy.

## Discussion and conclusions

Kidney involvement is common in SLE presenting with various pathologic patterns and clinical features. TMA, a rare but life-threatening complication of SLE, is characterised by endothelial injury that may cause thrombosis to the arterioles and capillaries. This results to microangiopathic haemolytic anaemia, thrombocytopaenia, and end-organ damage such as kidney impairment [7]. A retrospective study has shown that TMA development after SLE is more common than simultaneous occurrence of lupus nephritis and TMA [3]. The presence of TMA with SLE had poor renal outcomes despite conventional immunosuppressive drugs and plasmapheresis. The median overall survival in patients treated with vigorous immunosuppressive therapy and plasmapheresis was 2.9 months and 103.5 months in the concurrent (TMA-SLE) group and sequential (TMA development after SLE) group, respectively [8].

The patient was initially diagnosed with both TMA and SLE and; hence, it is a case of concurrent TMA-SLE. Despite recent advances regarding the mechanisms of TMA, a diagnosis of concomitant TMA in patients with SLE remains difficult as common clinical features of TMA, such as anaemia and thrombocytopaenia, are also frequently seen in SLE. Dysregulated complement activation plays an important role in the pathogenesis of SLE. This implies that SLE could unmask TMA by activating complement proteins in patients genetically predisposed to alternative complement pathway dysregulation [9]. Our case had TMA features consistent with lupus nephritis. Because the term aHUS usually applies to patients with genetic abnormalities, the patient was diagnosed with aHUS due to the presence of C3 mutation. Because TMA in SLE is associated with poor kidney outcome and short overall median survival, clinicians need to consider the possibility of aHUS in SLE patients for prompt diagnosis and treatment with eculizumab to improve renal recovery.

Recent genetic analysis has improved the understanding of aHUS pathogenesis [1]. Although mutations were found in up to 60 % of primary aHUS patients, these genetic defects are detected in only 5 % of patients with secondary HUS [10]. In a small study of complement-mediated TMA associated with lupus nephritis, complement-regulatory mutations were identified in 60 % of patients [11]. In the case, the patient was found to have a C3 gene mutation. In a French nationwide cohort, Fremeaux-Bacchi et al. has reported that 8.4 % of patients with aHUS had C3 mutations [12]. In a Korean registry, the frequency of C3 mutations was 8 % [13]. There were three reported cases of aHUS with C3-associated variants, specifically carrying the p.Ser562Leu mutations [13–15]. To our knowledge, this is the first case of co-existent lupus nephritis and aHUS with a C3 gene mutation.

Plasmapheresis is the first-line treatment for aHUS. However, response to treatment varies depending on the causative genetic abnormality. Since eculizumab has been introduced to treat primary aHUS [1], clinical outcomes have dramatically improved. However, the use of this complement inhibitor for the treatment of secondary HUS remains controversial [5, 6]. Recently, a case series has shown that eculizumab results to favourable outcomes in patients with aHUS and SLE [7, 11, 16]. Eculizumab led to improvement of symptoms, thrombocytopaenia, kidney function, and proteinuria in patients who were refractory to conventional immunosuppressive treatment and plasmapheresis [17].

These reports were consistent to the findings of the case. Eculizumab therapy on the patient who was refractory to plasmapheresis, corticosteroids, and cyclophosphamide resulted to the resolution of thrombocytopaenia with significant improvements on kidney function, and proteinuria. After four doses of eculizumab, platelet count doubled from baseline. After eight doses, urine albumin-to-creatinine ratio was halved. It appeared that eculizumab suppressed lupus activity since the autoantibody level was within the normal range during the course of therapy.

In summary, we report a unique case of co-existent lupus nephritis and C3 mutation-related aHUS that was successfully treated with eculizumab. Timely intervention with this complement inhibitor monoclonal antibody could be a convincing strategy for the treatment of severe lupus nephritis with co-existent TMA, the worst prognosis subtype.

## Data Availability

All data generated or analysed during this study are included in this published article.

## Abbreviations

aHUS: Atypical haemolytic uraemic syndrome
LDH: Lactate dehydrogenase
SLE: Systemic lupus erythematous
TMA: Thrombotic microangiopathy
